# Determinants of the mental health status of university students in Japan: comparison between pandemic and recession periods during the 7th wave of COVID-19

**DOI:** 10.3389/fpsyg.2023.1221333

**Published:** 2023-08-10

**Authors:** Yuko O. Hirano

**Affiliations:** Institute of Biomedical Sciences, Nagasaki University, Nagasaki, Japan

**Keywords:** mental health, university students, COVID-19 waves, recession, Japan

## Abstract

**Introduction:**

Although the number of identified coronavirus disease 2019 (COVID-19) cases and deaths has decreased, the repetitive crest-trough pattern of the spread of COVID-19 has yet to cease. The current situation may affect the mental health status of university students who are distracted in their academic and daily lives by the pandemic. This cross-sectional study aimed to identify the determinant factors of the mental health status of Japanese university students before and in the middle of the 7th wave of the pandemic, one of the largest waves to be experienced in Japan.

**Method:**

A survey was administered to two groups of students during the recession period between the 6th and 7th waves (*n* = 156) and during the 7th wave of the pandemic (*n* = 97).

**Results:**

*T*-tests showed no statistically significant differences between the two groups in Perceived Stress Scale (PSS) scores, while the COVID-19 anxiety scores (*p* < 0.001) and General Health Questionnaire (GHQ) scores were significantly higher in the Pandemic period group (*p* = 0.011). The multiple regression model indicated that PSS scores were the only determinant of GHQ scores in the Pandemic period group.

**Discussion:**

The results indicate that stressful life events, such as the COVID-19 pandemic and daily hassles, which are measured by the PSS, affect students’ mental health differently. Therefore, the author submits that school counselors should provide counseling to students to reduce anxiety caused by daily hassles, during recession and pandemic periods. Students also require assistance with the reduction of stress and anxiety caused by daily hassles, regardless of the pandemic situation.

## 1. Introduction

Global society has been influenced by the coronavirus disease of 2019 (COVID-19) since it was declared a pandemic on March 11, 2020 (World Health Organization, 2020a). Due to natural immunity from infection or immunity from vaccination (Fujii and Nakata, 2021; Wang et al., 2021; Zawbaa et al., 2022) and people’s increased health literacy regarding COVID-19 (Lopes and McKay, 2020; McCaffery et al., 2020), it seems that the COVID-19 pandemic is convergent as the number of confirmed cases and deaths has started to decrease (World Health Organization, 2023a). Many countries, including Japan, have resumed normal life again, as if COVID-19 no longer exists. Many Japanese universities resumed face-to-face classes by April 2022, and students have restarted their part-time jobs at shops or restaurants to earn an income. This has become a driving force for revitalizing economic activity in the country after the two-year interruption due to the pandemic. However, measures such as wearing a face mask, sanitizing hands, and avoiding the three Cs (closed spaces, crowded places, and close-contact settings) are still strongly recommended in Japan (Ministry of Health, Labour and Welfare, 2023a) because of the recurrent nature of the pandemic waves. As of March 2023, Japan has experienced more than 8 waves since the onset of the pandemic in early March 2020. Indeed, the wearing of face masks assists in the prevention of the transmission of the virus (Khan et al., 2020; Wiersinga et al., 2020; World Health Organization, 2020b). An individual’s choice to wear a mask is subject to epidemic conditions, health policies (Feng et al., 2020), and sociodemographic conditions (Sugimura et al., 2021). However, wearing a mask has been associated with anxiety symptoms (Wang et al., 2020), and this may be particularly true in Japan, where nearly 60% of the population wears masks (Fujii et al., 2021). Fujii et al. (2021) reported that Japanese people who wear masks are twice as likely to feel anxious that they will become infected with COVID-19 than those individuals who do not wear masks.

A long-lasting societal plight such as that caused by the COVID-19 pandemic may create an atmosphere that triggers the deterioration of mental health, which may lead to an increased risk for suicidal behavior. Sakamoto et al. (2021) reported that death by suicide has increased in Japan during the pandemic. This is particularly true for university students (Fuse-Nagase et al., 2021). However, few studies have been conducted to identify the determinants of maintaining good mental health, irrespective of whether people are in the midst of a pandemic wave. Such studies are especially important when governments are required to devise and implement measures to cope with repetitive pandemic waves. Previous studies have indicated that university students across the globe are more vulnerable to the influence of stressful events than older adults (Rossi et al., 2021). AlAteeq et al. (2020) found that university students in the Kingdom of Saudi Arabia experienced moderate to high levels of stress at the start of the COVID-19 outbreak. Amidst the pandemic, the mental health status of students has been affected by the sudden shift to e-learning classes (Fawaz and Samaha, 2021), unemployment confidence (Zheng et al., 2022), and uncertain future plans (Chowdhury et al., 2022). In addition, the perceived impact that university students believe that the COVID-19 pandemic will have on their lives has been reported to adversely affect their mental health. Aslan and Pekince (2021) reported that the perceived impact of COVID-19 on nursing students’ well-being was positively correlated with perceived stress and general anxiety disorder but negatively correlated with satisfaction with life. The perceived impact of COVID-19 has also been positively linked to perceived stress (Dymecka et al., 2023) in the general population and to depression (Simegn et al., 2021) in university students. With respect to the factors associated with the perceived impact of COVID-19 among university students and young adults, the sociodemographic environment, such as living together with family members, appears to contribute to lowering the psychological impact of COVID-19 (Sarsak, 2022). Social support is the most vital psychosocial protective resource to lower anxiety levels (Cao et al., 2020; Liu et al., 2020; Akbar and Aisyawati, 2021; Li et al., 2021) and depression (Liu et al., 2020; Li et al., 2021), while a lack of social support predicts increased negative mental health effects, such as anxiety and depression symptoms (Lai et al., 2020; Ma et al., 2020).

The ability to cope with stress, for example, by having a sense of coherence (SOC), has been associated with better psychological health and psychological well-being, as well as lower stress levels (Antonovsky, 1987). SOC comprises three elements, namely manageability, comprehensibility, and meaningfulness, which function as a resistance measure to daily stress. SOC is particularly relevant in coping with stress associated with the COVID-19 pandemic. For example, Shorey et al. (2022) reported that higher SOC scores were correlated with lower acute stress scores for nursing students in Southeast and East Asian Nursing Education and Research institutions during the pandemic. Similarly, Silva et al. (2021) found SOC was associated with better psychological well-being in undergraduate students in Brazil during the COVID-19 pandemic. However, most previous studies have focused on the impact of COVID-19 on the mental health of university students by using a cross-sectional design, with particular attention being paid to the pandemic period. Yet, pandemic waves can occur repeatedly because of variations in the virus. For instance, the 7th wave was caused by the omicron BA.5 variant, which hit Japan between early July and mid-September 2022, and was identified as the largest wave Japan had experienced in terms of the number of identified cases and deaths (National Institute of Infectious Diseases, 2022; Ministry of Health, Labour and Welfare, 2023b). This wave came after a 3-month decline during the convergence of the 6th wave, when people had resumed international travel behaviors driven by perceived knowledge about COVID-19 (Han et al., 2020).

It should be noted that, despite the number of infected patients during the 7th wave, many universities in Japan offered classes under the same conditions as during the recession period. Although infection prevention regulations, such as wearing masks during classes and sanitizing hands before entering classrooms, were implemented, the provision of student services, particularly psychological support services, to cope with the consequences of the pandemic, had not yet been properly established. To develop appropriate support for the mental health of students, the programs will need to be adaptable, because the psychological needs of the students may differ between pandemic and recession periods. In this study, the author aimed to identify the determinant factors of the mental health status of Japanese university students before and in the middle of the 7th wave, which can provide us basic data on the determinants of the mental health of university students according to the pandemic status.

## 2. Materials and methods

### 2.1. Participants

The participants were undergraduate students attending a national university in western Japan. The university has over 9,000 undergraduate and graduate students. There are eleven schools, including both medical (e.g., School of Medicine) and non-medical (e.g., School of Social Sciences, School of Environmental Science) schools. In order to select participants that were representative of the university, the author conducted stratified sampling by the following procedure. Considering the differences in health literacy, one medical department and three non-medical departments were selected using a 1:3 ratio, due to the distribution of students across departments.

The author originally planned to conduct a cross-sectional survey intermittently between June 15 and August 2, 2022, expecting to finish the study during the recession period. However, due to the unexpected emergence of the 7th wave, which arose in late July 2022, the author needed to statistically control for the influence of the pandemic on the participants. Therefore, the study participants were divided into two groups: the first group comprised students who completed the survey before the commencement of the 7th wave of the pandemic, and the second group comprised students who completed the survey during the 7th wave. The two groups were independent of each other, which allowed for a comparison of the impact of the 7th wave of the pandemic on the mental health of the students. The study was conducted between June 15 and August 2, 2022. According to the pandemic status database reported by the Ministry of Health, Labour and Welfare (2023b), the group that participated in the study between June 15 and July 1, 2022 was classified as the “Recession period group,” and those who participated on August 1 and 2, 2022 were classified as the “Pandemic period group.” The results obtained from the assessments explained in the next section were statistically compared between the groups.

### 2.2. Measures

A five-page questionnaire was created based on Lazurus and Folkman’s (1984) Transaction Model of Stress and Coping, which has been used in COVID-19-related studies (e.g., Jean-Baptiste et al., 2020; Osses-Anguita et al., 2023). Perceived impact of COVID-19 and perceived stress in daily life were assessed as independent variables that determine mental health outcomes. Social support and SOC were included as mediators of stress in the model. The same questionnaire was distributed to the participants of each group. It took 5 to 10 min to complete the questionnaire.

#### 2.2.1. General Health Questionnaire (GHQ)

This scale was utilized to examine the students’ mental health status. The author used the Japanese version of the GHQ-12, which was developed by Nakagawa and Daibou (2013). The GHQ, developed by Goldberg and Williams (1998) has been commonly used to screen for anxiety and depression in COVID-19-related studies (e.g., Kostić et al., 2021; Silva et al., 2021; Alam et al., 2022). The scale comprises 12 items relating to physical and mental conditions experienced in the preceding week. The participants were asked to respond using a 4-point Likert-type scale and choose how often they had experienced these physical and mental conditions. After reversing the 6 reverse-scored items, all 12 item scores were summed to give a total score (hereinafter, “GHQ score”). A higher GHQ score indicated a worse mental health status.

#### 2.2.2. COVID-19 anxiety score

Anxiety due to COVID-19 (hereinafter, “COVID-19 anxiety score”) was measured using a single item scale developed by the author, wherein the participants were asked, “How anxious are you that the COVID-19 pandemic may affect or has affected your daily life?” The participants were asked to respond using a five-point Likert-type scale, ranging from 1 = not at all to 5 = very much. The scale was developed for this study because there was no COVID-19-related anxiety scale for students. To develop the item for the scale, interviews were conducted with several students. Based on the interviews, it was determined that students tended to experience a general anxiety about the overall effect of COVID-19 on their daily lives. The convergent validity of the item was supported by its significant correlations with GHQ scores and Perceived Stress Scale (PSS) scores.

#### 2.2.3. Perceived Stress Scale (PSS)

This assessment tool was originally developed by Cohen et al. (1983) and comprises 10 items relating to minor stressors in daily life, such as embarrassment caused by unexpected events. The author adapted the PSS to measure daily hassles that may occur regardless of the pandemic conditions. The PSS assesses the frequency of occurrence of each item in the previous month (0 = never, 4 = very often). The PSS has been used as a measure of stress perception in other COVID-19-related studies (e.g., Aslan et al., 2020; Adjepong et al., 2022). In this study, the Japanese version of the PSS was used (Sumi, 2006).

#### 2.2.4. Social support

Perceived social support was measured by adapting 12 types of social support, including emotional and tangible support. The original scale was developed by Shima (1992) to measure the effect of social support in Japanese university students. Shima’s (1992) scale was the most suitable for this study in terms of the target population, university students, and the scale has been used in recent studies with university students in Japan (e.g., Xiao and Toyama, 2020; Hirano et al., 2022). The greater the number of types of social support, the more varied the perceived social support.

#### 2.2.5. Sense of Coherence (SOC)

Sense of coherence was used to investigate the participants’ stress resistance based on the theoretical framework developed by Antonovsky (1987). This theory emphasizes a salutogenic approach by providing a positive focus, identifying individual and collective resources, and by highlighting the importance of coherent measures and communication strategies (Mana and Sagy, 2020), which is useful to investigate profound challenges people must cope with, such as a pandemic. The SOC-3-UTHS, a Japanese version of the short-form SOC, was adapted for use in the present study (Togari et al., 2007). This self-reported questionnaire, which measures individuals’ SOC, can be divided into three subdomains: manageability, comprehensibility, and meaningfulness. The degree of agreement for each subdomain is rated on a 7-point Likert-type scale. The scores for each subdomain were added together to calculate the total SOC score. The higher the SOC score, the stronger the participants’ level of stress resistance. The author hypothesized that SOC may act as a buffer to mitigate the impact of stressors (COVID-19 anxiety score and PSS score) on mental health outcomes (GHQ).

#### 2.2.6. Sociodemographic characteristics

The questionnaire included items related to the following variables to assess sociodemographic characteristics, which were included as control variables in the multiple regression model. Gender was included because several epidemiological studies on mental health status during the COVID-19 pandemic indicated that women are more vulnerable than men in terms of mental health (Aslan et al., 2020; Tee et al., 2020; van der Velden et al., 2020; Kostić et al., 2021; Ochnik et al., 2021; Zhan et al., 2021; Adjepong et al., 2022; Kaltschik et al., 2022; Sarsak, 2022). Participants indicated which department they were enrolled in, which was used to control for academic major. Studies on the impact of COVID-19 have reported that stress levels (Horita et al., 2022), depression (Holm-Hadulla et al., 2021), and physical and mental health (Idris et al., 2021) varied according to students’ academic major. Participants also indicated their domicile status. Lai et al. (2020) reported that concern about the health of family or friends during the COVID-19 pandemic was positively associated with perceived stress and the severity of anxiety and depression symptoms. In this study, the participants were asked whether they lived alone.

### 2.3. Statistical analysis

Statistical analyses were performed using IBM SPSS Statistics Version 25-J. Bivariate analysis was performed using chi-square tests, *t*-tests, and Pearson’s correlation coefficient to test the association between variables. Multiple regression analysis was conducted to identify the factors indicating mental health status when the goodness of fit of the model of the determinants of mental health was tested. In the multiple regression analysis, the control variables, namely, gender, academic major, and domicile status, were included. Statistical significance was set at *p* < 0.05.

### 2.4. Ethical considerations

Informed consent was obtained from each participant prior to the distribution of the questionnaire, according to the Ethical Guidelines for Life Sciences and Medical Research Involving Human Subjects of Japan (Ministry of Health, Labour and Welfare, n.d.). The author and her research assistants explained the purpose of the study to the students face-to-face prior to obtaining their consent to participate. They were informed that participation in the study was voluntary and their responses to the questionnaire items would be held in confidence. This study was approved by the Biomedical Sciences Ethics Board of Nagasaki University (No. 23041801).

## 3. Results

The university in which the target population of this study was enrolled remained open throughout the study period, regardless of the pandemic conditions. Therefore, the author conducted the surveys without any constraints. Anonymous questionnaires were distributed to 302 students. A total of 254 questionnaires were returned (response rate: 84.1%): one hundred and fifty-seven (61.8%) from the Recession period group and 97 (38.2%) from the Pandemic period group. The participants included 101 male (39.8%) and 151 female (59.4%) students; two participants (0.8%) did not provide an answer to the gender question. Ninety-five participants (37.4%) were medical science majors, and 159 students (62.6%) were non-medical science majors. One hundred and seventy-seven students (69.7%) answered that they lived alone, while 76 (29.9%) lived with family members. The average age of the participants was 19.76 years (SD = 1.31).

The distribution of the participants’ characteristics between the Recession and Pandemic period groups was tested using chi-square tests. Gender (*p* < 0.001), academic major (*p* < 0.001), and domicile status (*p* = 0.001) differed significantly between the two groups. The Pandemic period group contained more women (79.4%), more medical science majors (74.2%), and fewer students who lived alone (58.3%) than the Recession period group.

The mean score of each main study variable is presented in Table 1. COVID-19 anxiety and GHQ scores were significantly higher in the Pandemic period group than in the Recession period group (*p* < 0.001, *p* = 0.011). The SOC score was significantly lower in the Pandemic period group than in the Recession group (*p* = 0.015). The correlation coefficients between the variables of the Recession period group are shown in Table 2, and those of the Pandemic period group are shown in Table 3. Strong correlation coefficients between PSS and GHQ scores were found in both the Recession (*r* = 0.533, *p* < 0.01) and Pandemic (*r* = 0.672, *p* < 0.01) period groups. Multiple regression analysis was conducted to identify the determinants of the mental health status of the participants by group. The mental health status of the Recession period group was significantly influenced by daily hassles (*β* = 0.457, *p* < 0.001) and social support (*β* = −0.369, *p* < 0.001). In the Pandemic period group, only daily hassles (*β* = 0.601, *p* < 0.001) significantly affected participants’ mental health status (Table 4).

**Table 1:** Distribution of the study variables by period group.

	Recession period group			Pandemic period group		
Scale variable	Range	Average	(SD)	Average	(SD)	Value of *p*
COVID-19 anxiety score	1–5	3.28	(1.14)	3.79	(0.94)	<0.001
PSS	6–36	20.63	(5.92)	21.84	(5.28)	0.102
Number of social supports	12–60	46.19	(10.25)	47.72	(7.53)	0.177
SOC	3–21	15.07	(3.07)	14.13	(2.71)	0.015
GHQ	12–41	25.73	(5.47)	27.60	(5.76)	0.011

PSS, Perceived Stress Scale; SOC, sense of coherence; GHQ, General Health Questionnaire; SD, standard deviation.

**Table 2:** Correlations among the study variables (recession period group).

Scale variable	COVID-19 anxiety score		PSS		Number of social supports		SOC		GHQ
COVID-19 anxiety score	1								
PSS	0.249	**	1						
Number of social supports	−0.130		−0.120		1				
SOC	−0.301	**	−0.350	**	0.316	**	1		
GHQ	0.168	*	0.533	**	−0.410	**	−0.284	**	1

PSS, Perceived Stress Scale; SOC, sense of coherence; GHQ, General Health Questionnaire.

**p < 0.01, *p < 0.05.

**Table 3:** Correlations among the study variables (pandemic period group).

Scale variable	COVID-19 anxiety score		PSS		Number of social supports		SOC		GHQ
COVID-19 anxiety score	1								
PSS	0.207	*	1						
Number of social supports	0.185		−0.192		1				
SOC	0.040		−0.299	**	0.253	*	1		
GHQ	0.158		0.672	**	−0.252	*	−0.287	**	1

PSS, Perceived Stress Scale; SOC, sense of coherence; GHQ, General Health Questionnaire.

**p < 0.01, *p < 0.05.

**Table 4:** Determinants of the mental health status of the students by period group.

Scale variable	Recession period group		Pandemic period group	
	*β*	Value of *p*	*β*	Value of p
COVID-19 anxiety score	−0.011	0.867	0.098	0.247
PSS	0.457	<0.001	0.601	<0.001
Number of social supports	−0.369	<0.001	−0.093	0.272
SOC	0.008	0.912	−0.102	0.227
Adjusted *R*^2^	0.388	<0.001	0.455	<0.001

Control variables: gender, academic major, and domicile status. PSS, Perceived Stress Scale; SOC, sense of coherence.

## 4. Discussion

In the current study, the author investigated the determinant factors of mental health among university students by dividing them into Pandemic period and Recession period groups, a topic not yet examined comprehensively. Surprisingly, the PSS score, and not the COVID-19 anxiety score, was the strongest predictor of mental health status, regardless of the period in which it was measured. The bivariate analysis indicates that there are some differences in the distribution of the independent variables, namely, COVID-19 anxiety and SOC, between the groups surveyed during the recession and in the middle of a pandemic wave. The threat of COVID-19 infection had less of an impact on the mental health status of the participants during the recession period. Indeed, the COVID-19 anxiety and GHQ scores were significantly higher in the Pandemic period group than in the Recession period group (Table 1). Nonetheless, the correlation coefficients between the COVID-19 anxiety and GHQ scores were very weak in both the Recession period (r = 0.168) and Pandemic period (*r* = 0.158) groups (Tables 2, 3). It is posited that as time passed, the participants became accustomed to the state of a “new normal” in which they had to co-exist with the virus and its mutations, which diminished the impact on their mental health status.

The number of newly confirmed cases in Japan in the 2 weeks prior to August 2, 2022 (the day the survey was terminated) was 1,234,282 (July 20 and July 26, 2022) and 1,434,864 (July 27 to August 2, 2022), respectively, which were the highest numbers since the start of the pandemic. This fact may be reflected in the COVID-19 anxiety score of the Pandemic period group, which was found to be significantly higher than that of the Recession period group. However, the COVID-19 anxiety score was not significantly associated with the GHQ score. Thus, despite the skyrocketing number of newly confirmed infections during the 7th wave, the participants in this study may not have perceived the threat of COVID-19 infection as being high for them. This interpretation is supported by information released by the Ministry of Health, Labour and Welfare (MHLW). According to the MHLW’s database, 201 severe cases of COVID-19 were reported between July 27 and August 2, 2022. Of these, 110 cases (54.7%) were found to be individuals older than 70 years, while only 6 cases (3.0%) were individuals in their 20s. The cumulative number of deaths reported in the same time period was 30,737. Of these, 26,731 cases (87%) were individuals older than 70 years, and 42 cases (0.001%) were individuals in their 20s (Ministry of Health, Labour and Welfare, 2023b). Therefore, the participants in this study may have had the perception that COVID-19 is not a major health threat to the younger generation. Consistent with this notion is the finding that Japanese younger generations are more willing to travel amid the 7th wave than older generations, and to use their own judgement about travelling as opposed to the measures adopted by the government (Travel Voice, 2022).

While a weak association was found between COVID-19 anxiety and GHQ scores, a strong association was apparent between PSS and GHQ scores. This was particularly true in the Pandemic period group. Pearson’s correlation coefficients indicated that the PSS score had the strongest correlations with the GHQ score in both the Recession and Pandemic period groups. Furthermore, a multiple regression model indicated that the PSS score was the only determinant factor of the GHQ score in the Pandemic period group. There are several possible explanations for why daily hassles was the strongest predictor of mental health status, especially in comparison with COVID-19 anxiety. As previously suggested, COVID-19 anxiety did not contribute significantly to the mental health status of the participants perhaps because they did not perceive the threat of COVID-19 as high for their age group. Second, daily hassles associated with being a university student may have outweighed any anxiety created by the pandemic. Daily hassles was also the strongest significant predictor during the recession period; it was just an even stronger predictor for students during the pandemic period. The pandemic may have exacerbated daily hassles, similar to what was found in Liu et al.’s (2022) study of U.S. mothers with children under the age of 9 years. Liu et al. (2022) also found a relationship between daily hassles and mental health (i.e., depression and anxiety) during the COVID-19 pandemic. Third, the anxiety that was assessed was anxiety caused by the pandemic in general. Brown and Harris (1978) stated that it can take just one stressful life event to cause an individual to develop depression (Brown, 2002). Therefore, if a more specific aspect of anxiety caused by the COVID-19 pandemic were assessed, for example, by asking “How anxious are you that you are not able to attend classes and will not be able to catch up due to the school being closed?” to assess how academic performance anxiety had been affected by the pandemic (Giusti et al., 2021; Horita et al., 2022), such specificity may have shown to be a stronger predictor of mental health.

This study’s results indicated that the determinants of mental health in the Recession period and Pandemic period groups were different. Even during the 7th wave of COVID-19, the COVID-19 anxiety score of the participants did not significantly determine their mental health status. This is assumed to be because the participants became accustomed to the COVID-19 living environment, and thus were likely to be less concerned about the risk of COVID-19 infection. Caution is required when applying the results of this study to students who were exposed to COVID-19 in the early stages of the pandemic, such as in the 1st or 2nd waves in 2020. Unlike in the early stages, the participants of the current study attended school as normal during the 7th wave. Therefore, one must be careful when comparing the results of this study with those of studies conducted in the earlier stages of the pandemic.

This study has some limitations. First, sampling bias was unavoidable. The participants attended classes at the time the survey was conducted; therefore, those who were absent from class because of COVID-19 could not be included in the study. Second, questions influencing the recognition of COVID-19 anxiety, such as anamnestic history of COVID-19 and/or other diseases, and vaccination history, were not included in the questionnaire. Third, a more precise assessment of COVID-19 anxiety that is unique to university students and its influence on academic performance should be employed in future studies.

Despite the above limitations, the author believes that the contributions of this study are substantial. It shows that even though more than 2 years have passed since the onset of the pandemic, students are still being influenced by COVID-19-related stressors. Concurrently, it indicates that students are becoming accustomed to the current conditions, and many are able to maintain a relatively normal life. The author concurs with the suggestion of Schiano di Cola et al. (2021), that the provision of school counselors to assist students is required, particularly in reducing stress caused by daily hassles, which may repeatedly occur. As the World Health Organization (2023b) warns, the world remains unprepared for another pandemic, and our attention must stay focused on measures to deal with future epidemics and pandemics or we shall pay a heavy price. The current study can provide basic data that are useful in the post-COVID-19 pandemic era, when repeated short waves of infection increase are expected. Further studies need to be conducted on how best to develop an effective intervention program for university students to help them maintain sound mental health in the pandemic era.

## Data availability statement

The raw data supporting the conclusion of this study will be made available by the author on reasonable request.

## Ethics statement

The studies involving human participants were reviewed and approved by Biomedical Sciences Ethics Board of Nagasaki University (No. 23041801). The patients/participants provided their written informed consent to participate in this study.

## Author contributions

The author confirms being the sole contributor of this work and has approved it for publication.

## Funding

This work was supported by Management Expenses Grants from Nagasaki University.

## Conflict of interest

The author declares that the research was conducted in the absence of any commercial or financial relationships that could be construed as a potential conflict of interest.

## Publisher’s note

All claims expressed in this article are solely those of the authors and do not necessarily represent those of their affiliated organizations, or those of the publisher, the editors and the reviewers. Any product that may be evaluated in this article, or claim that may be made by its manufacturer, is not guaranteed or endorsed by the publisher.
